# The Effect of Curcumin on TNF-α, IL-6 and CRP Expression in a Model of Polycystic Ovary Syndrome as an Inflammation State

**Published:** 2017

**Authors:** Shima Mohammadi, Parvin Kayedpoor, Latifeh Karimzadeh-Bardei, Mohammad Nabiuni

**Affiliations:** 1- Department of Animal Biology, Faculty of Biological Sciences, Kharazmi University, Tehran, Iran; 2- Laboratory’s Animal Center & Cellular and Molecular Research Laboratory, Faculty of Biological Sciences, Kharazmi University, Tehran, Iran; 3- Department of Cell and Molecular Biology, Faculty of Biological Sciences, Kharazmi University, Tehran, Iran

**Keywords:** CRP, Curcumin, Experimental model, IL-6, Immunohistochemistry, Inflammation, PCOS, TNF-α

## Abstract

**Background:**

Having low-grade chronic inflammation such as elevated C-reactive protein (CRP), interleukin-6 (IL-6) and tumor necrosis factor-α (TNF-α) plays a crucial role in polycystic ovary syndrome (PCOS). This study aimed at investigating the therapeutic effects of curcumin on IL-6, CRP and TNF-α and symptoms of polycystic ovary syndrome.

**Methods:**

In this research, 72 female adult Wistar rats were divided into control (n=12), PCOS (n=12) and curcumin-treated PCOS groups (n=48). PCOS was induced by injection of estradiol valerate (2 *mg/kg*-one-step). PCOS rats were divided into control and experimental groups which received daily intraperitoneal injection of curcumin. After 60 days of syndrome induction, ovaries were collected for histological and immunohistochemical evaluations. Serum IL-6 and CRP was detected by the ELISA kit. Data were analyzed using In-Stat 3 via one-way analysis of variance (ANOVA) and p<0.05 was considered statistically significant.

**Results:**

Histological studies showed a significant reduction in thickness of theca layer and increase in the number of corpus luteum (CL) diameter in the curcumin-treated group compared with the PCOS group; also inflammatory markers such as IL-6 and CRP significantly decreased in groups treated with curcumin compared with PCOS groups. Regarding immunohistochemical analysis, the expression of TNF-α in granulosa layer and follicular fluid of follicles and ovarian cysts in PCOS group was more than the control group’s expression. However, expression of this factor in the ovaries treated with curcumin was decreased.

**Conclusion:**

This study showed that the anti-inflammatory and antioxidant effects of curcumin on PCOS may be due to its inhibitory effect on expression and levels of TNF-α, serum IL-6 and CRP.

## Introduction

Polycystic ovary syndrome (PCOS) is a very common, heterogeneous endocrine disorder which accounts for about three-quarters of all cases of anovulatory infertility. Patients with PCOS also have metabolic disorders such as insulin resistance, dysfunction of beta cells in the pancreas, glucose intolerance, impaired lipid profile, and visceral obesity (1). The Rotterdam criteria were established to confirm diagnosis of PCOS in women who have at least two of the following symptoms; hyperandrogenism, polycystic ovaries and oligo- and/or anovulation (2). However, therapy and diagnosis of PCOS showed a uniform treatment pattern, and the current recommendations found in international literature have been widely implemented into clinical practice. In this study, PCOS was induced by estradiol valerate (EV). Administration of EV to adult rats leads to anovulation and cystic ovarian morphology. Studies showed that administration of EV during adult reproductive life disrupts cyclicity via activation of the sympathetic ovarian nerve and increased ovarian expression of norepinephrine (NE). Sympathetic ovarian nerve denervation (SONX) overcomes this disruption (3, 4). On histopathological assessment, polycystic ovaries contain increased numbers of follicles, hypertrophy and luteinization of the inner theca cell layer, and a thickened ovarian tunica (5). Some studies on abnormal endocrine parameters like high insulin resistance, have indicated increasing concentrations of LH (Luteinising hormone), and hyperandrogenism, which may be associated with reduced fertilization rate and abnormal embryonic development (6). Many studies have confirmed that in women with PCOS, a positive relationship exists among CRP values and insulin resistance, body weight and fatty mass. CRP, IL-6, IL-18 and TNF-α have been related to the risk of developing type 2 diabetes and cardiovascular diseases that are strongly associated with insulin resistance and body fat amount (7). Among the cytokines, IL-6 is a regulator of inflammation and immunity, modulating the secretion of various cytokines, and promoting T-cell activation and B-cell differentiation (8). In patients with PCOS, circulating levels of TNF-α, IL-6, and CRP as well as white blood cell (WBC) count and neutrophil count have been found to be elevated compared with age- and/or body mass index (BMI)-matched controls (9). IL-6 plays an important role in ovarian maturation and implantation process. IL-1β and TNF-α are important markers that influence ovulation and pregnancy. IL-1 secreted in follicle can regulate granulosa cells to synthesize prostaglandin and control the collagenase activity, which play a crucial role in ovulation (10). TNF-α is an inflammatory cytokine that stimulates and proliferates follicular theca cells; also, it plays an important role in regulating the normal activities of ovaries in follicular and lutein development stage. TNF-α is detected in macrophages, oocytes, granulosa cells, atresia, and theca cells. It has been shown that overexpression of this cytokine in human and rodent adipose tissue causes insulin resistance and rise in glucose levels (11).

Curcumin is a yellow polyphenol extracted from the rhizome of turmeric (Curcuma longa), a plant grown tropical Southeast Asia (12). Recent experimental evidence demonstrated an anti-inflammatory, anti-diabetic, and anti-obesity role for curcumin in mouse models of obesity and diabetes (13). Studies have shown that increased calcium in cytosol may be due to an increase in reactive oxygen species (ROS).On the other hand, curcumin may inhibit the entry of calcium and protein kinase C through the effect anti-angiogenic and antioxidant (14). It can modulate the activation of T-cells, B-cells, macrophages, neutrophils, natural killer (NK) cells, and dendritic cells. These molecular evidences suggest that favorable effects on cancers might be due to direct anti-oxidative and anti-inflammatory effects, as well as the ability to modulate the immune systems (15). By inhibiting growth factor of vascular endothelial cells (VEGF), its specific receptor and angiogenesis, curcumin stops angiogenesis and prevents the formation of new blood vessels in tumor cells and their growth (16).

Considering the importance of expression changes of TNF-α signaling, IL-6 and CRP levels and also the effects of anti-inflammatory, antioxidant and anti-fibrotic of curcumin, expression of these cytokines was measured in samples of syndrome treated with curcumin compared with PCOS groups in this study.

## Methods

### Animals:

A total number of 72 female Wistar rats, weighing approximately 170±20 *gr* were used in the current study. The animals were kept under standard laboratory conditions (12 *hr* light/dark cycle, 26–28°*C*) which were preserved until the end of the investigation. Keeping the animals under these situations had started 1 week before the study. Animal cages were kept clean, and food and water were given regularly every day. All experiments in this study were performed by observing the guidelines for animal research issued by the National Institutes of Health and were approved by the Local Committee on Animal Research (National Research Council, 1996).

### Study design and treatment:

The rats were divided into three groups: the control group (n=12), the PCOS group (n=12) and the experimental (treated) group. For PCOS induction, the experimental group received 2 *mg/kgBW* estradiol valerate (Aboureihan, Iran) by intramuscular injection and in one step (17). After 60 days, using chloroform 3, rats were deeply anesthetized in order to ensure the induction of PCOS. Induction in rats was compared with the control. The control group received no injection. To determine the appropriate amount of curcumin, the concentration of a substance that causes death in 50% of mice was considered LD50 (that is defined as the concentration of lethal dose causing death in 50% of mice), which in this probe, 2000 *mg/kgBW* curcumin was determined as the concentration of LD50. Curcumin is a lipophilic polyphenol and thus is insoluble in water, but it is readily soluble in organic solvents such as dimethyl sulfoxide (DMSO). A stock solution of curcumin (Sigma) was prepared at 100 *mmol/L* in DMSO. Therefore, concentrations of 100, 200, 300, 400 *mg/kg* of body weight were selected as the treatment. After 14 consecutive days of treatment with curcumin, rats were killed by chloroform inhalation and ovarian samples were isolated. After getting ovary out of the rat body and removing excess tissues for histologic report and histochemistry techniques, it was placed in Bouin’s Fixative and paraformaldehyde.

### IL-6 assay:

According to the manufacturer’s instructions, serological analysis was performed to measure serum IL-6 levels and hormonal alterations. IL-6 was determined by an ELISA (rat IL-6 platinum ELISA®, Bender Medsystems, Austria). All samples were analyzed in one assay.

### Histological analysis:

After molding with paraffin, ovarian samples were cut into slices with a diameter of 6–7 *mm* and placed on glass slides coated with gelatin. Then, tissue samples were stained with hematoxylin–eosin and finally the number of follicle was examined by light microscopy at different stages of development and follicular layer thickness.

After stabilization of ovarian samples, all three groups were kept in paraformaldehyde for 12 *hr* for qualitative evaluation of immunohistochemistry. The steps to the sample preparation in the investigation of the histology were similar until cutting stage. The only difference was glass slides used for immunohistochemistry that were coated with 0.1% polylysine. After placing the slides in incubator for 24 *hr*, paraffin was removed with xylene and it was taken with descending grades of alcohol, tissue sections for antigen retrieval, respectively in 10 *mM* citrate buffer with pH=6, were put for 20 *min* at temperature 90°*C*. Then, to remove non-specific binding sites of the primary antibody, it was rinsed with PBS buffer water. Then, the slices with diluted TNF-α polyclonal antibody (50:1) 4% PBS-BSA (Phosphate-buffered saline-Bovine serum albumin) for 24 *hr* at 4°*C* were incubated in wet chambers. After washing in PBS, by placing the slides in a solution of hydrogen peroxidase 0.3% in methanol for 10 *min*, inhibition of cell peroxidase activation happened. Next, the slides were washed and incubated with secondary antibody for TNF in one hour at room temperature in a humidified chamber. Detection was performed by the diaminobenzidine (DAB) dye and using immunohistichemistry accessory kit and forming a brown precipitate. Data analysis was carried out by using a light microscope (Zeiss, Germany). For statistical analysis, image J, one-way ANOVA (Analysis of Variance) with post-hoc Tukey HSD (Honestly Significant Difference) test were used and p-value less than 0.05 was considered significant and the corresponding graphs were plotted using EXCEL program.

The H-score system was based on the proportion and intensity of brown staining cells. For H-score assessment, ten fields were chosen at random at 400× magnification and the staining intensity of each slide was scored as 0, 1, 2 or 3 corresponding to the presence of negative, weak, intermediate or strong brown staining, respectively. The total number of cells in each field and the number of cells stained at each intensity were counted. The average percentage positive was calculated and the following formula was applied: H-Score=(% of cells stained at intensity 1×1)+(% of cells stained at intensity 2×2)+(% of cells stained at intensity 3×3). H-score between 0 and 300 was obtained where 300 was equal to 100% of tumor cells stained strongly (18).

## Results

To investigate the morphological changes in rats, the animals in both the control and the PCOS group were weighted and then the results were examined. In addition to a significant increase in body weight of PCOS group compared to the control group (p<0.001), the increase in abdominal fat was also observed macroscopically compared to PCOS group. After intraperitoneal injection of curcumin for 14 days, abdominal fat tissue was modulated and reduced in body weight (p<0.001) (Table 1).

**Table 1.** Hormone concentrations, ovarian and body weight of rats in the control, PCOS and treated with curcumin groups (n=12). In all groups, control was compared with PCOS group and curcumin treated PCOS was compared with PCOS group

**Groups**	**Ovarian weight (*mg*)**	**Body weight (*g*)**	**E2 (*ng/ml*)**	**T (*ng/ml*)**	**LH (*ng/ml*)**	**P4 (*ng/ml*)**	**FSH (*ng/ml*)**
**Control**	12.08±0.5	160.3±8.46	0.27±0.01	0.73±0.03	2.36±0.15	66.03±7.7	1644.76±126.6
**PCOS**	20.4±0.15**	217.6±15***	0.63±0.05 ***	1.41±0.3*	8.16±1.56***	31.12±1.76 **	476.82±13.3***
**Cucumin100 *mg/kg BW***	17.4±0.39***	207±16.25***	0.36±0.04	0.8±0.03	4±0.1***	35.69±1.07**	431.13±29.81***
**Cucumin200 *mg/kg BW***	15.4±0.15	196±10.78***	0.3±0.01	0.8±0.01	3.1±0.04***	47.36±0.88*	613.24±13.05***
**Cucumin300 *mg/kg BW***	14.3±0.2	189.20±8.36***	0.29±0.01	0.77±0.02	2.56±0.09	50.33±0.41	1174.05±15.95**
**Cucumin400 *mg/kg BW***	12.9±0.17	184.28±5.9***	0.26±0.01	0.7±0.01	2.39±0.01	53.95±0.2	1591.21±44.97

E2: 17β-estradiol; T: Testosterone; LH: Luteinizing Hormone; P4: Progesterone; FSH: Follicle Stimulating Hormone; PCOS: Polycystic Ovary Syndrome.

*** p<0.001,

** p<0.01,

* p<0.05

After killing the rats, taking blood from the heart, abdominal incisions and peritoneum, an attempt was made to remove the ovaries, superfluous fat and oviduct tubes and then the ovaries were weighed on sensitive scales (SARTORIUS-Germany). The mean of ovarian weight in PCOS group significantly increased compared to control group (p<0.01). Increasing the level of follicular fluid and ovarian stroma could lead to ovarian weight gain in the PCOS compared to the control group. However, the mean of ovarian weight in the treated samples (100 *mg/kgBW*) decreased in contrast to the PCOS group (p<0.001) (Table 1).

The analysis of LH in PCOS and treated groups with curcumin (100 and 200 *mg/kgBW*) compared with the control group (p<0.001) was done, but at other doses of curcumin (300 and 400 *mg/kgBW*), decreased LH serum levels showed no significant change compared with the PCOS group. Compared to the experimental group, testosterone and sex hormone level in PCOS showed significant difference (p<0.05, p<0.001) with curcumin. Regarding the result of the current study, testosterone and estradiol level in the experimental group did not show significant differences compared to the PCOS group, but a sharp decline was reported. Evaluation of changes of FSH and the hormone progesterone showed a significant increase (p<0.001, p<0.01, p<0.05) between the curcumin treated-PCOS and PCOS group compared with the control group (Table 1).

**Figure 1.**
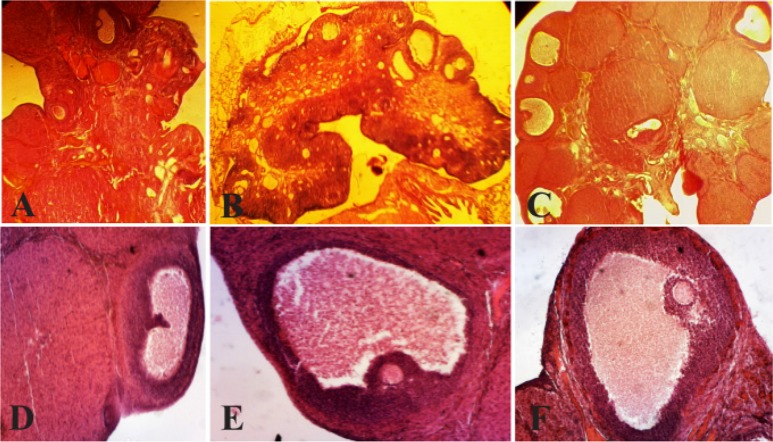
Histological analysis of normal ovaries (A, D) compared with PCOS (B, E) and ovaries treated with curcumin (C, F). The morphological changes of the rats’ ovarian tissues were stained with hematoxylin and eosin, as described in the Materials and Methods section. A, D) A representative rat’s ovarian tissue section from the control group, which had normal appearance (A×100,40). B, E) A representative rat’s ovarian tissue section from the PCOS group showed thickening surface albuginea, under which there were many follicles in different phases (including atretic follicles and cystic dilating follicles), as well as fewer layers of granular cells, disappeared oocytes and corona radiating within the follicles (B×100, 40). C, F) A representative rat’s ovarian tissue section from the group treated with curcumin, which showed increased granular cell layers, and some ovulation phenomena (C×100, 40) (Scale bar, 50 *μm* A, B, C), (Scale bar, 20 *μm* D, E, F). AF: atretic follicle, CF: cystic follicle (_*_), CL: corpus luteum, GCL: granular cell layer (Δ), TCL: theca cell layer (→)

In order to determine the effect of curcumin on follicular development, regarding morphology, follicles were classified into six groups: 1- primordial follicles (PMF), 2- primary follicles (PF), 3- pre-antral follicles, less than 600 *μ* (PAF), 4- antral follicles, 600 to 1000 *μ* (AF), 5- cystic follicles (CF) and 6- corpus luteum (CL). In the ovaries of the PCOS, a large number of small follicles and large cystic follicles with a thin granulosa layer were observed, which is the characteristic of PCOS. On the other hand, corpus luteum was not observed in the PCOS group. In the control group, there was no ovarian cyst and corpus luteum was abundant indicating normal ovulation in this group. Therefore, reported observation confirmed the creation of the cyst and stopping of follicular development after 60 days of treatment with estradiol valerate, due to lack of normal ovulation, cyst formation and normal follicular growth distribution (Figure 1).

After treatment with curcumin, morphological studies showed that in a low amount of curcumin (100 *mg/kgBW*), a number of cysts with various sizes were observed. Raising the amount of curcumin (200, 300, 400 *mg/kgBW*) in the treated ovaries, no cysts were observed and also the number of corpus luteum was increased which marked the beginning of ovulation. In PCOS group injected with curcumin (100 *mg/kgBW*), a significant increase in number of corpus luteum was observed compared with PCOS group (p<0.001). In PCOS group treated with curcumin, the number of cysts and antral follicles was significantly reduced in comparison with PCOS group (p<0.001, p<0.01). In all groups treated with curcumin (200, 300, 400 *mg/kgBW*), PCOS group manifested significant increase in the number of primordial follicles, preantral, and corpus luteum (Table 2).

**Table 2.** Morphometry of groups follicular and ovarian follicular diameter (*μm*) (n=12). In all groups, control was compared with PCOS group and curcumin treated PCOS was compared with PCOS group

**Groups**	**PMF**	**PF**	**PAF**	**AF**	**CF**	**CL**	**Follicle diameter**
**control**	45±0.1	20±0.1***	29±0.1***	18±0.1***	0±0***	10±0.1*	554±0.8***
**PCOS**	42±0.05	12±0.05	17±0.05	3±0.15	17±0.1	4±0.1	722±0.3
**Cucu100 *mg/kg***	48±0.1***	14±0.1	20±0.15	9±0.15**	11±0.1***	18±0.1***	667±0.4***
**Cucu200 *mg/kg***	51±0.05***	15±0.11***	25±0.1***	10±0.11***	10±0.1***	19±0.1***	621±0.3***
**Cucu300 *mg/kg***	56±0.15***	19±0.5***	45±0.15***	11±0.1***	7±0.1***	21±0.2***	611±0.05***
**Cucu400 *mg/kg***	66±0.2***	21±0.1***	46±0.1***	14±0.05***	1±0.1***	25±0.1***	598±0.2***

In polycystic ovary groups treated with curcumin, a significant increase in the number of follicle and decrease in the ovaries treated with low amount of curcumin was observed in all groups (except group of primordial follicles). In addition, there was a significant decrease in the number of ovarian cysts. PMF: Pri-Mordial Follicles; PF: Primary Follicles: PAF: Pre-Antral Follicles; AF: Antral Follicles; CF: Cystic Follicles; CL: Corpus Luteum.

*** p<0.001,

** p<0.01,

* p<0.05

The diameter of follicles in the PCOS ovaries had a significant increase in the size of the follicles among control group and PCOS group (p< 0.001). There was a significant decrease in follicle size between the groups treated with curcumin (100 *mg/kgBW*) and PCOS group (p<0.001). Treatment with higher value of curcumin (200, 300 and 400 *mg/kgBW*) despite the reduction in the size of the follicles was not considered significant (Table 2).

### Changes in CRP:

In this study, CRP changes in PCOS induced group as shown in table 3 caused a significant increase in the level compared with the control group. In the group treated with curcumin (100, 200, 400 *mg/kgBW*), systemic inflammation marker CRP decreased significantly compared with the PCOS group (p<0.001) (Table 3).

**Table 3.** The mean of H-score, IL-6 and CRP compared to the control group, PCOS and treated with curcumin (n=12)

**Groups**	**Stroma**	**Preantral**	**Grannulosa**	**Theca**	**CRP (*pg/ml*)**	**IL-6 (*pg/ml*)**
**Control**	115±2.6	12±2	11±3	10±2	0.175±.09*	0.597±0.008***
**PCOS**	137±2.5*	55±3.2***	117±3.5***	14±2.1***	0.325±.007	0.771±0.006
**Cucu100 *mg/kg***	114±2.5	60±3	89±3.5***	12±2*	0.26±.01***	0.769±0.007
**Cucu200 *mg/kg***	92±4.1***	45±2	57±5***	16±2.3***	0.179±.005***	0.679±0.002***
**Cucu300 *mg/kg***	105±2**	51±1	13±2***	9±1.9	0.115±.006*	0.6±0.02***
**Cucu400 *mg/kg***	101±1.5**	38±2.9*	22±2.5***	10±2	0.065±.04***	0.594±0.01***

*** p<0.001,

** p<0.01,

* p<0.05

### IL-6 assay:

In this study, PCOS induction led to a significant rise in IL-6 inflammatory index (p< 0.001 *vs*. control rats). The effect of curcumin on the level of IL-6 in PCOS rats was examined for 14 days after comparison with the PCOS group. The respective IL-6 levels in control group, PCOS and curcumin-treated PCOS rats (100, 200, 300, 400 *mg/kgBW*) were 0.76, 0.67, 0.60 and 0/59 *pg/ml*. The IL-6 level in curcumin-treated rats (200, 300 and 400 *mg/kgBW*) was reduced. Results showed that administration of curcumin significantly reduced the IL-6 level in comparison with PCOS group (Table 3).

### Immunolocalization of α-TNF:

**Figure 2.**
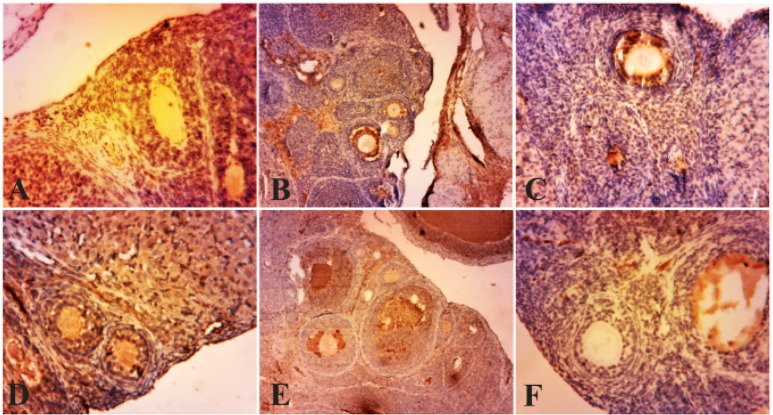
Photo micrographs of immunohistochemical expression of TNF-α in the primary and secondary follicles. (A) normal ovary; (B) strict expression of TNF-α is observable in these follicles in PCOS group; (C, D, E, F) TNF-α expression is restricted to blood vessels and ovary stroma (filled arrow) in curcumin groups (100, 200, 300, 400 *mg/kg BW*). Magnification ×100, (Scale bar, 25 *μm*)

**Figure 3.**
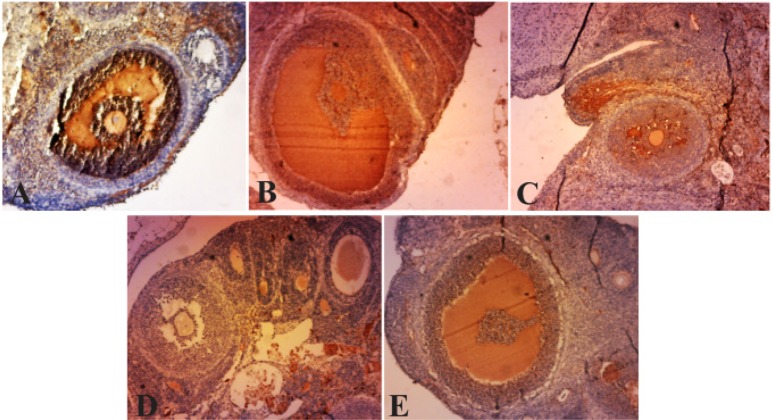
Photo micrographs of immunohistochemical expression of TNF-α in infolded layers and follicular fluid of Graafian follicles and cysts. A) Due to high levels of angiogenesis, the density of follicular fluid, as well as TNF-α expression are high. The thick theca and granulosa layers indicate high expression levels of TNF-α in PCOS group. B, C, D, F) decreased TNF-α expression levels in follicular fluid and granulosa layer in curcumin groups (100, 200, 300, 400 *mg/kgBW*), Magnification ×40, (Scale bar, 20 *μm*). Granular cell layer (→), follicular liquid of Graafian follicle (Δ)

Considering the importance of the expression of TNF-α in stimulating, proliferation and steroidogenesis in the ovarian follicle theca cells, this study examined the expression levels of TNF-α, in pre-antral and antral follicles which were observed in the granulosa layer in PCOS ovaries. But in normal ovaries and the ones treated with curcumin, the expression of this factor in the granulosa layer of antral and pre-antral follicles was rarely compared to PCOS group (Figure 2). This enzyme is not expressed in the follicular theca but in follicular fluid, a brown precipitate is obvious. Expression levels of TNF-α in several layers of ovarian follicular cells treated with curcumin showed reduction compared with PCOS group while granulosa layers in the pre-antral, antral follicles, and the cells surrounding the oocyte expressed less TNF-α (Figure 3).

In PCOS group, strong immunoreactivity to TNF-α was observed in ovarian stroma, preantral follicles, and granulosa layer (p<0.001, p<0.05). Weak TNF-α expression was seen in theca layer (p<0.001) compared with the control group. TNF-α expression in stromal cell in the curcumin-treated PCOS group showed significant decrease (p<0.001, p<0.01, p<0.05) in comparison with PCOS. Also, immunostaining in the theca layer in curcumin-treated PCOS group was less intense than PCOS group (p<0.001, p<0.05). In curcumin-treated PCOS group compared with PCOS group, TNF-α presented low expression in granulosa and preantral follicles (p<0.001, p<0.05) (Table 3).

## Discussion

The overall aims of treatment for patients with PCOS are to induce ovulation for women desiring to conceive, to reduce androgen levels, body weight and long-term health risks of diabetes mellitus and cardiovascular disease (19). In general, most women with PCOS have multiple small follicles in the ovary, which often quickly respond to injectable gonadotropin medications (20). In 2003, Yildirim et al. suggested that the main peripheral and visceral fat thickness increases in PCOS. Women with PCOS have abnormalities in the metabolism of androgens and estrogen production (21). Natali et al. suggested that the granulosa cells in follicular cysts of PCOS in women may be abnormal. In contrast, the strong staining seen with these methods in the theca layer, could be explained by the carbohydrates associated with the increased amount of collagen (22). The results of this study showed an increase in the thickness of the theca layer and a reduction in the thickness of granulosa cell layer in PCOS samples. It also confirmed a reduction in theca layer thickness and an increase in granulosa cells in patient samples treated with curcumin. Generally, increasing the amount of visceral fat led to an increase in the amount of TNFα in serum. Anna et al. reported that serum level of TNF-α was shown to increase in patients with PCOS, and may concur to determine a condition of low-grade inflammation eliciting an overproduction of IL-6 monocytes following microbial inflammatory stimulus (23). In order to investigate the possible relationship between IL-6, hyperandrogenemia and anthropometric or metabolic alterations on the one hand, and to gain further insight into the significance of PCOS related endocrine and metabolic abnormalities, both IL-6 and CRP as parameters of chronic inflammation in PCOS rats were measured.

Matthews et al. in 2001 showed that C-reactive protein (CRP) is associated with insulin resistance and as such increases both the risk of development of diabetes type 2 and cardiovascular risk (24). Hector et al. in 2011 explained that CRP was significantly higher in PCOS patients. An analysis of the most comparable studies indicates elevated circulating CRP in PCOS which suggests disordered chronic low-grade inflammation. They also found that elevated circulating CRP in PCOS is independent from obesity since this finding has persisted after excluding all the studies with mismatches in frequency of obesity or BMI between groups from the analysis (25). Citing the researches done by Velija-Asimi in 2006, reduction in CRP was consistent with a decrease in metabolic symptoms of PCOS subjects who were treated with metformin. Curcumin treatment was also associated with a decrease in CRP levels and these findings along with the findings of other histological and molecular studies are in line with the therapeutic effects of curcumin on the syndrome (26). The result of this study is in line with those performed by Heutling et al. in 2006. They showed that the levels of proinflammatory cytokines (IL-6 and IL-18) were significantly increased in women with PCOS and were found to be positively correlated with parameters of insulin sensitivity; treatment with metformin positively affected metabolic and endocrine profile as well as menstrual function and led to spontaneous pregnancies (27). *In vitro* studies have shown the ability of proinflammatory stimuli to upregulate the steroidogenic enzyme responsible for androgen production in ovarian theca cells (28). Cytokines can also stimulate proliferation of theca cells. In addition, abnormal inflammatory input may impair the finely tuned inflammatory processes used to control ovulation. In that light, the excess cytokine gene expression in the ovary of IR/LepR mice (Insulin resistance and leptin receptor) is interesting (29). In 2009, Schaaf showed that curcumin has anti-proliferative, pro-apoptotic and hormone suppressive effects on pituitary tumor cells. These effects were not only observed in pituitary tumor cell lines *in vitro* and *in vivo* but also, to a lesser extent, in human pituitary adenoma cells as well. Therefore, curcumin by itself, or in combination with already used drugs may be a promising tool for the development of new pharmacological treatment concepts of pituitary tumors (30). In 2014, Stephanie et al. stated the inhibition of phosphorylated JNK (c-Jun N-terminal kinases), p38 and ERK1/2 (Extracellular signal-regulated kinase 1/2) signalings which can be one of the molecular mechanisms which induced curcumin inhibition of the regulation of IL-6 mRNA expression in LPS (Lipopolysaccharide) vascular smooth muscle cells *in vitro* (31). Adipose tissue-derived cytokine expression (TNF-α, IL-6) may be an important contributor to low grade chronic inflammation. In other words, accumulation of visceral adipose tissue may be a key factor that underpins features of the metabolic syndrome and low grade chronic inflammation (32). In 2008, Miller et al. stated that curcumin inhibits the proliferation of tumor cells in the pituitary gland, induces apoptosis and reduces the production and release of hormones. So, they suggested that treatment with curcumin can be used as a new treatment (33). In this study, anti-inflammatory aspects of curcumin were investigated. Curcumin by affecting the size of polycystic ovary and minimizing it macroscopically will affect the number of primordial follicles and corpus luteum and reduce them after treatment. Gulcubuk et al. in 2013 suggested that reductive effect of curcumin in tissue damage could be occurred by inhibition of trypsin and NF-kB (Nuclear factor kappa-light-chain-enhancer of activated B cells) activation and hence induction of apoptosis. However, curcumin decreased tissue damage and the lack of direct correlation among tissue damage, amylase, lipase, free oxygen radicals and proinflammatory cytokine levels in some subgroups remained in this study as an issue to be thoroughly investigated; thus further studies are needed (34). The immunohistochemical study of polycystic ovaries treated with curcumin showed a significant decrease in the levels of TNF-α and probably this factor plays an important role in production of cysts and the pathogenesis of polycystic ovary syndrome.

## Conclusion

This study showed that curcumin via inhibition of TNF-α and IL-6, by reducing the follicular sheath, improving ovulation and corpus luteum, will improve histologic features of polycystic ovary and will push it towards having active and healthy ovary.

## References

[1] Amer SAK. Polycystic ovarian syndrome: diagnosis and management of related infertility. Obstet Gynaecol Reprod Med. 2009;19(10):263–70.

[2] Broekmans FJ Knauff EA Valkenburg O Laven JS Eijkemans MJ Fauser BC. PCOS according to the Rotterdam consensus criteria: Change in prevalence among WHO-II anovulation and association with metabolic factors. BJOG. 2006;113(10):1210–7. 16972863 10.1111/j.1471-0528.2006.01008.x

[3] Abbott DH Dumesic DA Eisner JR Colman RJ Kemnitz JW. Insights into the development of polycystic ovary syndrome (PCOS) from studies of prenatally androgenized female rhesus monkeys. Trends Endocrinol Metab. 1998;9(2):62–7. 18406243 10.1016/s1043-2760(98)00019-8

[4] Lara HE Ferruz JL Luza S Bustamante DA Borges Y Ojeda SR. Activation of ovarian sympathetic nerves in polycystic ovary syndrome. Endocrinology. 1993;133(6):2690–5. 7902268 10.1210/endo.133.6.7902268

[5] Hart R Hickey M Franks S. Definitions, prevalence and symptoms of polycystic ovaries and polycystic ovary syndrome. Best Pract Res Clin Obstet Gynaecol. 2004;18(5):671–83. 15380140 10.1016/j.bpobgyn.2004.05.001

[6] Järvelä IY Sladkevicius P Kelly S Ojha K Campbell S Nargund G. Quantification of ovarian power Doppler signal with three-dimensional ultrasonography to predict response during in vitro fertilization. Obstet Gynecol. 2003;102(4):816–22. 14551013

[7] Repaci A Gambineri A Pasquali R. The role of low-grade inflammation in the polycystic ovary syndrome. Mol Cell Endocrinol. 2011;335(1):30–41. 20708064 10.1016/j.mce.2010.08.002

[8] Daraï E Detchev R Hugol D Quang NT. Serum and cyst fluid levels of interleukin (IL) -6, IL-8 and tumour necrosis factor-alpha in women with endo-metriomas and benign and malignant cystic ovarian tumours. Hum Reprod. 2003;18(8):1681–5. 12871882 10.1093/humrep/deg321

[9] Tarkun I Arslan BC Cantürk Z Türemen E Sahin T Duman C. Endothelial dysfunction in young women with polycystic ovary syndrome: relationship with insulin resistance and low-grade chronic inflammation. J Clin Endocrinol Metab. 2004;89(11):5592–6. 15531516 10.1210/jc.2004-0751

[10] Xu X Du C Zheng Q Peng L Sun Y. Effect of metformin on serum interleukin-6 levels in polycystic ovary syndrome: a systematic review. BMC Womens Health. 2014;14:93. 25096410 10.1186/1472-6874-14-93 PMC4149309

[11] McGrath KC McRobb LS Heather AK. Androgen therapy and atherosclerotic cardiovascular disease. Vasc Health Risk Manag. 2008;4(1):11–21. 18629352 PMC2464747

[12] Aggarwal BB Sundaram C Malani N Ichikawa H. Curcumin: the Indian solid gold. Adv Exp Med Biol. 2007;595:1–75. 17569205 10.1007/978-0-387-46401-5_1

[13] Ejaz A Wu D Kwan P Meydani M. Curcumin inhibits adipogenesis in 3T3-L1 adipocytes and angiogenesis and obesity in C57/BL mice. J Nutr. 2009;139(5):919–25. 19297423 10.3945/jn.108.100966

[14] Maheshwaria RK. Singha AK Gaddipatia J Srimal RC. Multiple biological activities of curcumin: a short review. Life Sci. 2006;78(18):2081–7. 16413584 10.1016/j.lfs.2005.12.007

[15] Basnet P Skalko-Basnet N. Curcumin: an anti-inflammatory molecule from a curry spice on the path to cancer treatment. Molecules. 2011;16(6):4567–98. 21642934 10.3390/molecules16064567 PMC6264403

[16] Thangapazham RL Sharma A Maheshwari RK. Multiple molecular targets in cancer chemoprevention by curcumin. AAPS J. 2006;8(3):E443–9. 17025261 10.1208/aapsj080352 PMC2761050

[17] Karimzadeh L Nabiuni M Kouchesfehani HM Adham H Bagheri A Sheikholeslami A. Effect of bee venom on IL-6, COX-2 and VEGF levels in polycystic ovarian syndrome induced in Wistar rats by estradiol valerate. J Venom Anim Toxins Incl Trop Dis. 2013;19(1):32. 24330637 10.1186/1678-9199-19-32 PMC4029518

[18] Kalantari MR Nazeran T Varshoee Tabriz F. Quick score and H-Score assessment of P504s (AMACR) expression in Renal Cell Carcinoma (RCC) and relation with histologic grade. Iran J Pathol. 2012;7(5):157–64.

[19] Wang HS. The role of metformin in the treatment of polycystic ovary syndrome (PCOS). Chang Gung Med J. 2006;29(5):445–7. 17214387

[20] Egbase PE Al-Sharhan M Grudzinskas JG. ‘Early coasting’ in patients with polycystic ovarian syndrome is consistent with good clinical outcome. Hum Reprod. 2002;17(5):1212–6. 11980740 10.1093/humrep/17.5.1212

[21] Yildirim B Sabir N Kaleli B. Relation of intra-abdominal fat distribution to metabolic disorders in nonobese patients with polycystic ovary syndrome. Fertil Steril. 2003;79(6):1358–64. 12798883 10.1016/s0015-0282(03)00265-6

[22] Salvetti NR Gimeno EJ Lorente JA Ortega HH. Expression of cytoskeletal proteins in the follicular wall of induced ovarian cysts. Cells Tissues Organs. 2004;178(2):117–25. 15604534 10.1159/000081721

[23] Fulghesu AM Sanna F Uda S Magnini R Portoghese E Batetta B. IL-6 serum levels and production is related to an altered immune response in polycystic ovary syndrome girls with insulin resistance. Mediators Inflamm. 2011;2011:389317. 10.1155/2011/389317 PMC3086286 21547256

[24] Matthews DR Hosker JP Rudenski AS Naylor BA Treacher DF Turner RC. Homeostasis model assessment: insulin resistance and beta-cell function from fasting plasma glucose and insulin concentrations in man. Diabetologia. 1985;28(7):412–9. 3899825 10.1007/BF00280883

[25] Jatzko B Ott J. Circulating inflammatory markers in polycystic ovary syndrome: a systematic review and meta-analysis. Fertil Steril. 2011;96(4):e158. 21839436 10.1016/j.fertnstert.2011.07.1098

[26] Velija-Asim Z. Metformin decreases CRP level and cardiovascular risk in PCOS women. Endocr Abstracts. 2006;11:705.

[27] Heutling D Schulz H Nickel I Kleinstein J Kaltwasser P Krzyzanowska K Endothelial, inflammatory and endocrine markers in women with PCOS before and after metformin treatment. Exp Clin Endocrinol Diabetes. 2006;114(S 1):P15–195.

[28] Ortega I Stener-Victorin E Villanueva JA Sokalska A Stanley SD Letrozole increases growth of rat theca-interstitial cells and Cyp17a1 gene expression in the rat ovary. Fertil Steril. 2011;96(7):447–59. 10.1016/j.fertnstert.2012.11.006 PMC3667963 23200686

[29] Marino JS Iler J Dowling AR Chua S Bruning JC Coppari R Adipocyte dysfunction in a mouse model of polycystic ovary syndrome (PCOS): evidence of adipocyte hypertrophy and tissue-specific inflammation. PLoS One. 2012;7(10):e48643. 23119079 10.1371/journal.pone.0048643 PMC3485364

[30] Schaaf C Shan B Buchfelder M Losa M Kreutzer J Rachinger W Curcumin acts as anti-tumorigenic and hormone-suppressive agent in murine and human pituitary tumour cells in vitro and in vivo. Endocr Relat Cancer. 2009;16(4):1339–50. 19726538 10.1677/ERC-09-0129

[31] Stephanie Y Yuliani FG Winarno T Suhartono M. Inhibition of interleukin-6 expression by curcumin in rat vascular smooth muscle explants in vitro. Am J Biochem Biotechnol. 2014;10(4):260–6.

[32] Festa A D’Agostino R Jr Howard G Mykkänen L Tracy RP Haffner SM. Chronic subclinical inflammation as part of the insulin resistance syndrome: the Insulin Resistance Atherosclerosis Study (IRAS). Circulation. 2000;102(1):42–7. 10880413 10.1161/01.cir.102.1.42

[33] Miller M Chen S Woodliff J Kansra S. Curcumin (diferuloylmethane) inhibits cell proliferation, induces apoptosis, and decreases hormone levels and secretion in pituitary tumor cells. Endocrinology. 2008;149(8):4158–67. 18450960 10.1210/en.2007-1760 PMC2488238

[34] Gulcubuk A Haktanir D Cakiris A Ustek D Guzel O Erturk M Effects of curcumin on proinflammatory cytokines and tissue injury in the early and late phases of experimental acute pancreatitis. Pancreatology. 2013;13(4):347–54. 23890132 10.1016/j.pan.2013.05.005

